# I’m Completely Satisfied But…: Barriers and Facilitators to Fulfilling Nursing Home Residents’ Preferences

**DOI:** 10.1093/geroni/igaa057.3064

**Published:** 2020-12-16

**Authors:** Caroline Madrigal, Liza Behrens, Vanessa Burshnic, Julie Murpy, Katherine Abbott, Kimberly Van Haitsma

**Affiliations:** 1 Providence VA Medical Center, Providence, Rhode Island, United States; 2 University of Pennsylvania School of Nursing, Philadelphia, Pennsylvania, United States; 3 Durham VA Health Care System, Durham, North Carolina, United States; 4 Philadelphia, Pennsylvania, United States; 5 Miami University, Oxford, Ohio, United States; 6 Penn State University, University Park, Pennsylvania, United States

## Abstract

The US federal government mandates nursing homes (NHs) provide preference-based, person-centered care for residents. Yet only 2% of NHs successfully implement person-centered care. The Care Preference Assessment of Satisfaction (ComPASS) is a 16-item tool that facilitates residents’ preference fulfillment (i.e., satisfaction with quality of daily care and activities). This study examines barriers/facilitators to preference fulfillment from the perspective of residents who completed ComPASS. Secondary data analysis from an R21 (NR011334-01) provided qualitative data from 196 residents across 28 NHs on why residents were satisfied (or not) with the fulfillment of their individual preferences. Most residents were female (70.4%) and white (80.1%) with a range of cognitive/physical abilities. Content analysis revealed six thematic codes classifying barriers/facilitators to preference fulfillment: resident agency, values, and physical characteristics; social support systems; staff competence; communication success; built environment; access to resources. Discussion will include implications for ameliorating barriers to preference fulfillment while meeting government mandates. Part of a symposium sponsored by the Research in Quality of Care Interest Group.

